# Establishment of a Seven-Gene Signature Associated with CD8^+^ T Cells through the Utilization of Both Single-Cell and Bulk RNA-Sequencing Techniques in Clear Cell Renal Cell Carcinoma

**DOI:** 10.3390/ijms241813729

**Published:** 2023-09-06

**Authors:** Yubin Chen, Xinyu Zhou, Yanwei Xie, Jianan Wu, Tingting Li, Tian Yu, Yipeng Pang, Wenlong Du

**Affiliations:** 1 Department of Bioinformatics, School of Life Sciences, Xuzhou Medical University, Xuzhou 221004, China; c2380901384@163.com (Y.C.); zxy34018@163.com (X.Z.); 19551296011@163.com (Y.X.); wujianan0212@163.com (J.W.); 19802652626@163.com (T.L.); 19852208878@163.com (T.Y.); yipengpangxzhmu@163.com (Y.P.); 2 Department of Biophysics, School of Life Sciences, Xuzhou Medical University, Xuzhou 221004, China

**Keywords:** CD8^+^ T cells, prognosis, clear cell renal cell carcinoma, immunotherapy, drug responses

## Abstract

Tumor immune microenvironment constituents, such as CD8^+^ T cells, have emerged as crucial focal points for cancer immunotherapy. Given the absence of reliable biomarkers for clear cell renal cell carcinoma (ccRCC), we aimed to ascertain a molecular signature that could potentially be linked to CD8^+^ T cells. The differentially expressed genes (DEGs) linked to CD8^+^ T cells were identified through an analysis of single-cell RNA sequencing (scRNA-seq) data obtained from the Gene Expression Omnibus (GEO) database. Subsequently, immune-associated genes were obtained from the InnateDB and ImmPort datasets and were cross-referenced with CD8^+^ T-cell-associated DEGs to generate a series of DEGs linked to immune response and CD8^+^ T cells. Patients with ccRCC from the Cancer Genome Atlas (TCGA) were randomly allocated into testing and training groups. A gene signature was established by conducting LASSO-Cox analysis and subsequently confirmed using both the testing and complete groups. The efficacy of this signature in evaluating immunotherapy response was assessed on the IMvigor210 cohort. Finally, we employed various techniques, including CIBERSORT, ESTIMATE, ssGSEA, and qRT-PCR, to examine the immunological characteristics, drug responses, and expression of the signature genes in ccRCC. Our findings revealed 206 DEGs linked to immune response and CD8^+^ T cells, among which 65 genes were correlated with overall survival (OS) in ccRCC. A risk assessment was created utilizing a set of seven genes: *RARRES2*, *SOCS3*, *TNFSF14*, *XCL1*, *GRN*, *CLDN4*, and *RBP7*. The group with a lower risk showed increased expression of CD274 (PD-L1), suggesting a more favorable response to anti-PD-L1 treatment. The seven-gene signature demonstrated accurate prognostic prediction for ccRCC and holds potential as a clinical reference for treatment decisions.

## 1. Introduction

Among the genitourinary cancers, renal cell carcinoma (RCC) is the leading cause of death. Around 90% of renal tumors are identified as clear cell renal cell carcinoma (ccRCC) [1]. According to statistics, approximately 75% of individuals diagnosed with RCC can expect to survive for a minimum of 5 years, whereas individuals with advanced ccRCC face a considerably lower 5-year survival rate of 11.2% [2]. The incidence of ccRCC is on the rise in developed societies [3]. While surgical intervention is a successful approach for treating ccRCC in its initial stages, the outlook for patients with advanced disease is grim [4]. Timely detection plays a pivotal role in enhancing patient outcomes for ccRCC [5]. There remain no efficacious biomarkers available in clinical settings for the early detection of ccRCC [6]. Therefore, it is essential to develop reliable biomarkers that can assist in the prompt detection and surveillance of ccRCC advancement [7].

Among the notable attributes of tumors is an imbalanced immune tumor microenvironment (TME) [8]. The TME encompasses diverse cellular constituents, such as tumor cells, stromal cells, and immune cells [9]. CD8^+^ T cells, as the primary effector cells against tumors in the TME, primarily function as cytotoxic cells. However, the effectiveness of their role is impeded by the existence of cells and molecules that suppress the immune system [10]. As the immune response weakens, there is an observed increase in the expression of checkpoint genes, such as CTLA4 and PD-1, on the surface of CD8^+^ T cells [11]. Hence, acquiring a thorough comprehension of the regulation of CD8^+^ T cells in the TME is of utmost importance.

Earlier studies have shown that the infiltration of CD8^+^ T cells in tumors is indicative of prognosis in ccRCC and is linked to an adverse clinical result [12,13]. However, immune checkpoint blockade (ICB) and TME-modulating drug treatment were found to benefit ccRCC patients with more infiltrating PD1^+^, CD8^+^ T cells, and Treg^+^ immune cells [14]. It is worth mentioning that among the immune cells in ccRCC, CD8^+^ T cells account for the majority [15]. Moreover, the downregulation of SNHG1 has been reported to enhance the infiltration of CD8^+^ T cells in ccRCC mice, leading to prolonged overall survival (OS) [6]. This unconventional finding may be attributed to the diverse functional condition and variability of CD8^+^ T cells observed in ccRCC. To date, our comprehension of the regulatory mechanisms and clinical significance of CD8^+^ T cells in ccRCC remains incomplete. Consequently, a more profound understanding of the immune-associated genes that govern CD8^+^ T cells has the potential to unveil novel targets for immunotherapy in ccRCC [16].

The objective of this research was to employ scRNA-seq and bulk RNA-seq data acquired from TCGA in order to detect genes linked to CD8^+^ T cells and establish a predictive risk model in ccRCC. The results of this research could potentially enhance the prediction of prognosis and ameliorate treatment outcomes for patients with ccRCC, especially regarding the response to immunotherapy.

## 2. Results

### 2.1. Identification of Immune- and CD8^+^ T-Cell-Related DEGs

**Figure 1:**
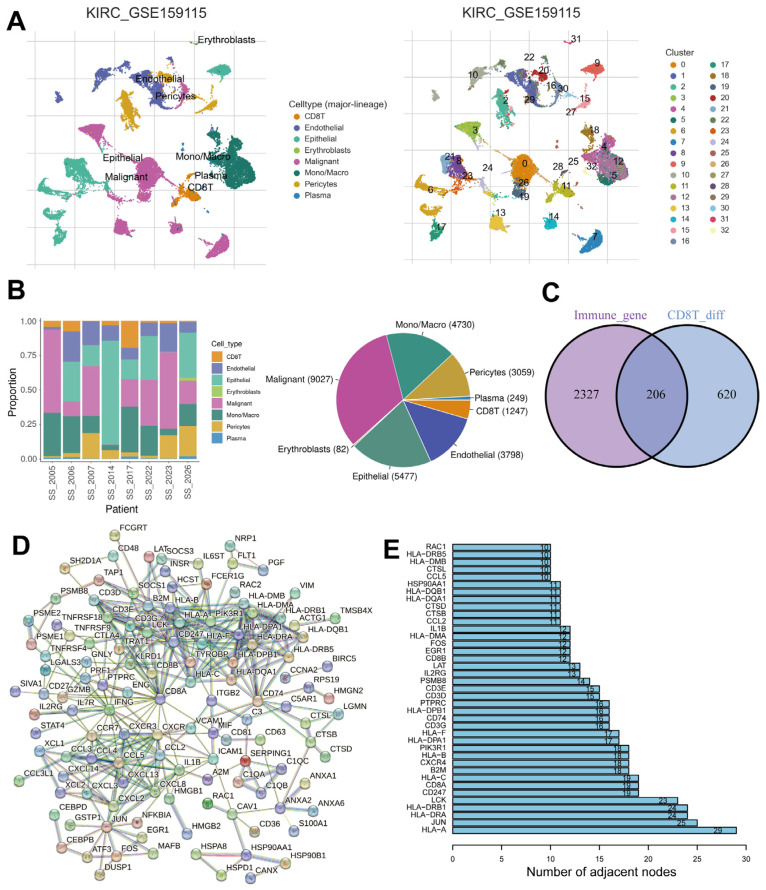
Identification of immune- and CD8^+^ T-cell-related DEGs from the TISCH Database. (**A**) Annotation and clustering of all cell types in GSE159115. (**B**) The proportion and number of each cell type for eight patients. (**C**) A Venn diagram of immune-associated genes and DEGs related to CD8^+^ T cells. (**D**) The PPI network of common targets for immune-associated genes and DEGs related to CD8^+^ T cells. (**E**) Histogram showing the number of adjacent nodes.

The GEO database (GSE159115) was utilized to examine scRNA-seq data from eight ccRCC patients, aiming to uncover the heterogeneity of ccRCC tissue. By employing dimensional reduction and clustering analysis techniques, we successfully identified 8 distinct cell types and 33 cell clusters within the ccRCC tissue (Figure 1A). Notably, Figure 1B illustrates the varying proportions of these eight cell types across different patient tissues. Erythroblasts constituted the smallest fraction, whereas malignant cells constituted the largest proportion. In order to identify DEGs associated with immune response and CD8^+^ T cells, we initially filtered 826 DEGs from a gene matrix comparing CD8^+^ T cells to other cell types (fold change > 1.5, *p*-adjust < 0.05). Subsequently, we performed additional filtering by matching 2533 immune-associated genes from the InnateDB and ImmPort datasets with DEGs, resulting in a final set of 206 immune- and CD8^+^ T-cell-related DEGs (Figure 1C, Supplementary Table S1). To elucidate the interplay among these DEGs, we created a network of protein–protein interactions (PPI) which included the 206 DEGs related to immune response and CD8^+^ T cells (Figure 1D). Notably, the PPI network revealed a subset of hub genes that ranked within the top 39 nodes (Figure 1E).

### 2.2. Construction of a Seven-Gene Signature Related to CD8^+^ T Cells in ccRCC

**Figure 2:**
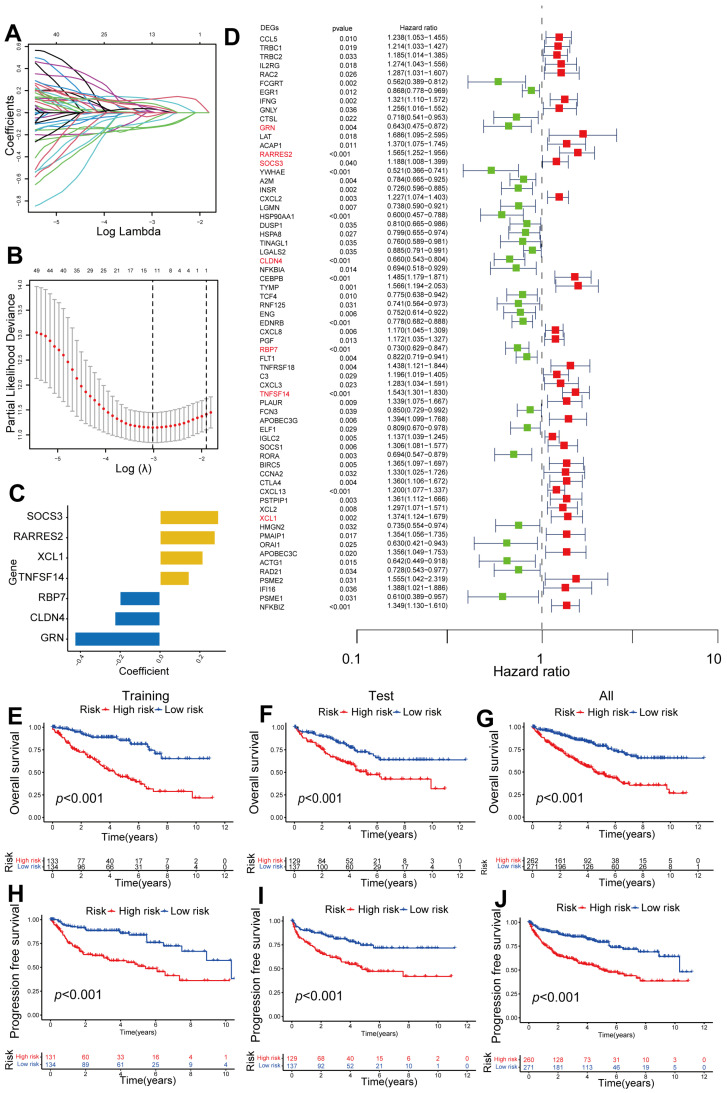
Construction of a 7-gene signature related to CD8^+^ T cells. (**A**) An analysis of 65 CD8^+^ T-cell-associated genes using the LASSO coefficient. (**B**) A 10-time cross-validation procedure was utilized to tune arguments in the LASSO model. (**C**) A barplot showing the coefficients of the 7 CD8^+^ T-cell-associated genes. (**D**) Univariate analysis of 65 CD8^+^ T-cell-related genes. The 7 CD8^+^ T-cell-associated genes utilized to construct the risk model are highlighted in red. (**E**–**G**) OS analysis of ccRCC patients with high risk and low risk in the training, testing, and overall sets. (**H**–**J**) Progression-free survival (PFS) analysis of ccRCC patients with high risk and low risk in the training, testing, and overall sets.

Based on the 206 DEGs related to CD8^+^ T cells, a univariable Cox proportional hazards regression analysis was conducted to determine the relationship between them and the prognosis of ccRCC. Among the 206 genes, a total of 65 genes were discovered to have a strong correlation with overall survival (OS) in the TCGA cohort (*p* < 0.05, Figure 2D). These 65 survival-related genes were then utilized for identifying prognostic genes to construct risk predictive models using LASSO regression. Through this analysis, seven genes were identified as powerful prognostic factors (Figure 2A,B), and the coefficients of each factor can be found in Figure 2C. In order to evaluate risk, we created a gene signature according to these seven risk prediction factors. A factor coefficient formula was utilized to count the risk score: Risk score = (−0.428 × *GRN*)  +  (0.277 × *RARRES2*)  +  (−0.295 × *SOCS3*)  +  (−0.227 × *CLDN4*)  +  (−0.201 × *RBP7*)  +  (0.147 × *TNFSF14*)  +  (0.217 × *XCL1*). The samples were classified into high-risk and low-risk groups based on the median value of their risk scores. A total of 533 patients with ccRCC were randomly assigned to either the training set (*n* = 267) or the testing set (*n* = 266). Table 1 presents a comparison of the clinical characteristics of these two patient sets, indicating no significant clinical differences between the training and testing groups. Notably, the group with high risk exhibited considerably poorer OS rates (Figure 2E–G) and progression-free survival (PFS) rates across the training, testing, and overall groups (Figure 2H–J), in comparison to the group with low risk.

### 2.3. Validating the Prognosis of CD8^+^ T-Cell-Associated Genes in Training, Testing, and Overall Datasets

**Figure 3:**
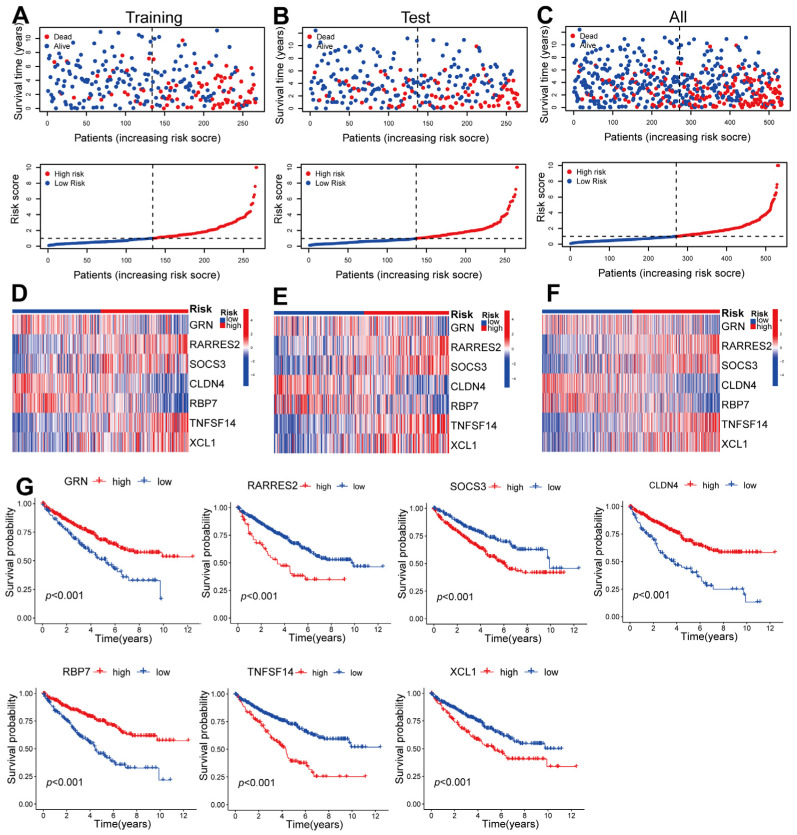
Verification of the prognosis of CD8^+^ T-cell-associated genes was conducted in the training, testing, and overall datasets. (**A**–**C**) Dot plots were generated to illustrate the survival and risk score for the training, testing, and overall cohorts. (**D**–**F**) Gene expression heatmaps were constructed to compare the expression levels of the 7 CD8^+^ T-cell-associated genes between the high- and low-risk groups in the training, testing, and entire cohorts. (**G**) KM analysis was performed to assess the impact of high and low expression of these 7 CD8^+^ T-cell-related genes on ccRCC patients.

**Figure 4:**
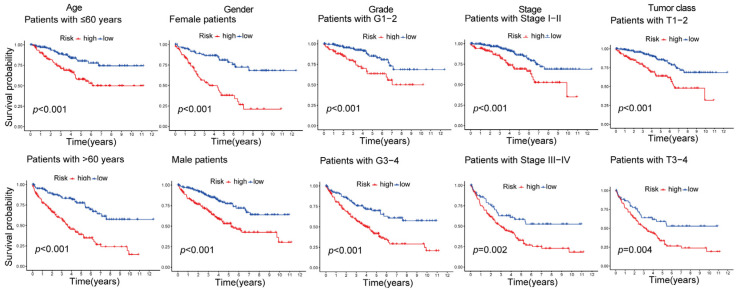
Kaplan-Meier (KM) survival plots for individuals with varying clinical factors, categorized as low and high risk.

For the purpose of survival analysis, patients in the training set were categorized into groups at high and low risk based on their scores with respect to median risk. The mortality rate of patients with ccRCC increased proportionally to their risk scores (Figure 3A). This phenomenon consistently occurred in both the testing and entire sets (Figure 3B,C). Notably, *RARRES2*, *SOCS3*, *TNFSF14*, and *XCL1* exhibited significantly stronger expression levels in the high-risk set compared with the low-risk set across the training, testing, and entire datasets (Figure 3D–F). These findings suggest that these genes, which are associated with CD8^+^ T cells, could potentially function as significant predictors of unfavorable outcomes. On the other hand, the research unveiled that the expression levels of *GRN*, *CLDN4*, and *RBP7* were considerably decreased in the high-risk category when compared to the low-risk category. This finding suggests that these specific genes related to CD8^+^ T cells may have the potential to serve as protective indicators, as depicted in Figure 3D–F. Furthermore, the results obtained from the Kaplan–Meier (KM) analysis provided additional confirmation that these seven CD8^+^ T-cell-associated genes are closely related to the prognosis of ccRCC (Figure 3G). Additionally, an examination was undertaken to compare the survival probabilities of ccRCC patients based on a variety of factors, including age, gender, stage, and G and T stage. Irrespective of the evaluation of clinical variables, it was observed that individuals classified as high risk experienced a shorter OS compared with those classified as low risk (Figure 4). This finding implies that the model we built is effectively applicable to diverse clinical parameters.

### 2.4. Independent and Superior Prognostic Ability of the Seven-Gene CD8^+^ T-Cell-Associated Signature in Patients with ccRCC

**Figure 5:**
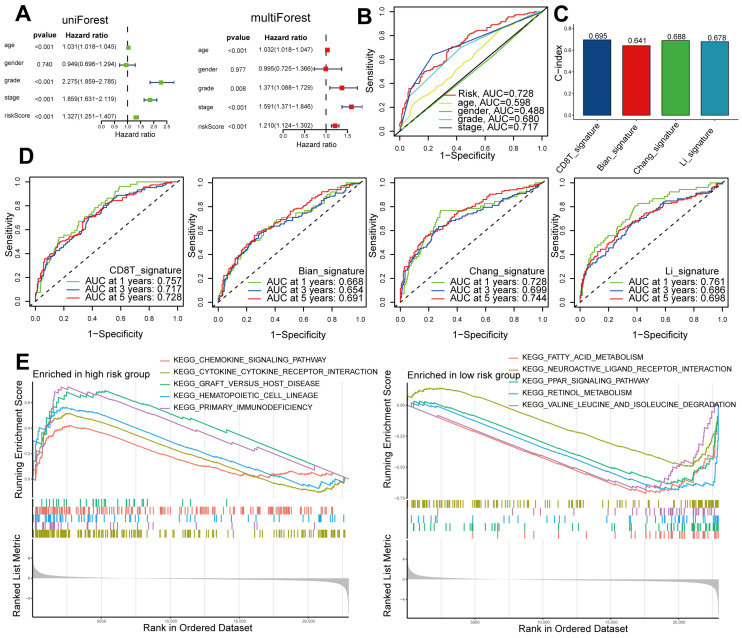
Independent and superior prognostic ability of the 7-gene CD8^+^ T-cell-related signature in patients with ccRCC and a comparison of high- and low-risk sets using GSEA. (**A**) Clinical characteristics and risk scores were analyzed using both univariate and multivariate methods. (**B**) AUC values were calculated using the risk score and additional clinical variables. (**C**) Comparison of our risk signature with others based on the C-index. (**D**) Receiver operating characteristic (ROC) curves of our risk signature compared with the others. (**E**) GSEA showcasing the pathways that are most enriched for sets classified as high risk and low risk.

For patients diagnosed with ccRCC, both univariate and multivariate Cox regression analyses demonstrated that the risk score serves as an independent prognostic factor (Figure 5A). Notably, the risk score exhibited superior predictive capability compared to other clinical characteristics, as evidenced by an area under the curve (AUC) of 0.728 (Figure 5B). To assess the performance of our signature, we selected several signatures from previous studies (Bian, Chang, and Li) [17,18,19], and we employed the same methodology to calculate their respective risk scores. Our signature, CD8T, slightly outperformed these comparative signatures in terms of AUC at 3 years, as well as the C-index (Figure 5C,D). In our comparative analysis of models, we noticed that our model demonstrated exceptional performance in prognostic prediction. To determine the enriched gene sets between high-risk and low-risk patients, we employed Gene Set Enrichment Analysis (GSEA). The group at high risk displayed enrichment in gene sets associated with chemokine signaling, cytokine receptor interaction, graft-versus-host disease, hematopoietic cell lineage, and primary immunodeficiency. Conversely, the low-risk group exhibited enrichment in gene sets related to fatty acid metabolism, neuroactive ligand receptor interaction, PPAR signaling, retinol metabolism, and valine, leucine, and isoleucine degradation (Figure 5E).

### 2.5. Comparing DEGs Associated with High- and Low-Risk Patients Based on Functional Enrichment

**Figure 6:**
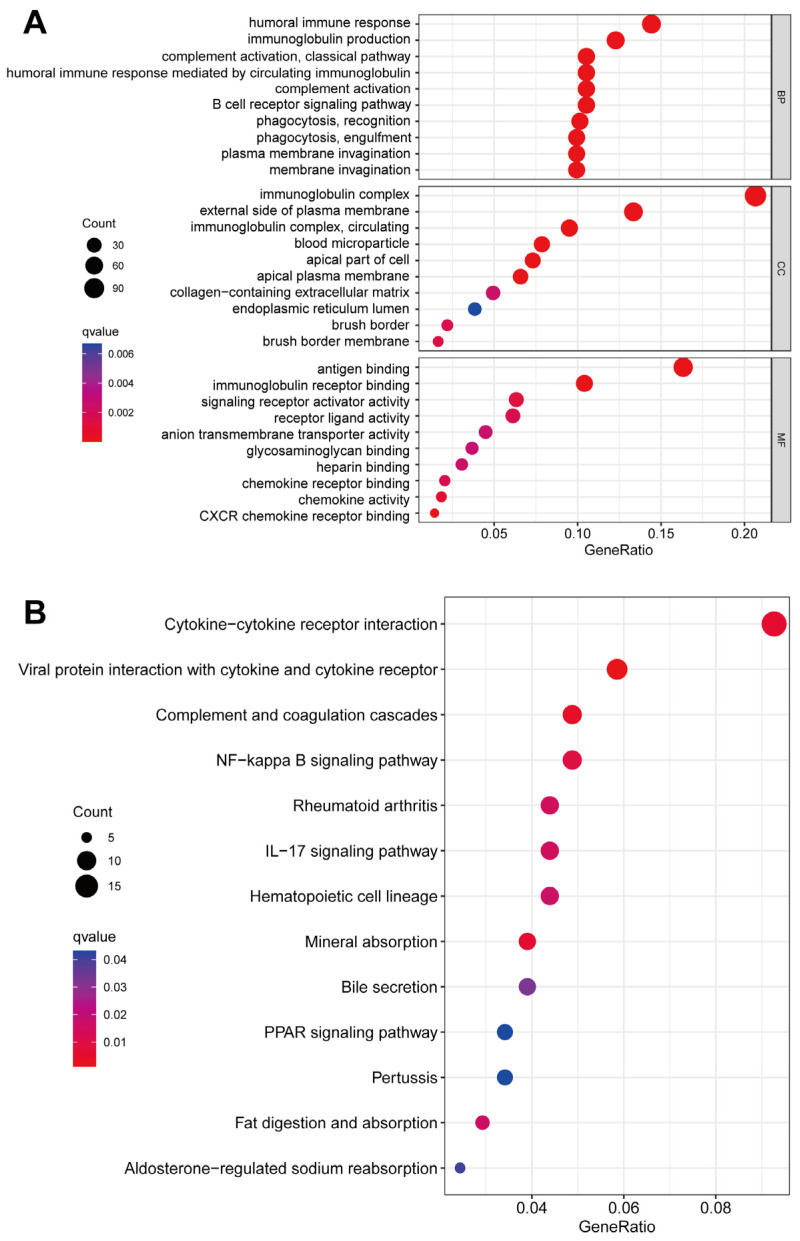
An analysis of GO and KEGG data between patients at high risk and those at low risk. (**A**) In the GO analysis, a diversity of biologic processes (BPs), cellular components (CCs), and molecular functions (MFs) were identified. (**B**) According to KEGG analysis, significant pathways were identified.

In order to gain a deeper comprehension of the potential biological mechanisms underlying the DEGs associated with high- and low-risk patients, we performed an analysis utilizing the GO and KEGG databases. The investigation revealed that the biological processes primarily encompassed the humoral immune response, immunoglobulin production, complement activation, and phagocytosis. Additionally, the molecular functions were found to be associated with antigen binding, immunoglobulin receptor binding, signaling receptor activator activity, and receptor ligand activity. The cellular components analyzed in this study encompassed the immunoglobulin complex, the external side of the plasma membrane, blood microparticles, and the apical part of the cell (Figure 6A, Supplementary Table S2). Through KEGG analysis, it was determined that these DEGs were primarily associated with cytokine–cytokine receptor interaction, viral protein interaction with cytokine and cytokine receptor, complement and coagulation cascades, and the NF-kappa B signaling pathway (Figure 6B, Supplementary Table S3).

### 2.6. Risk-Score-Based Characteristics of ccRCC Tumor Mutations and Response to Chemotherapy

**Figure 7:**
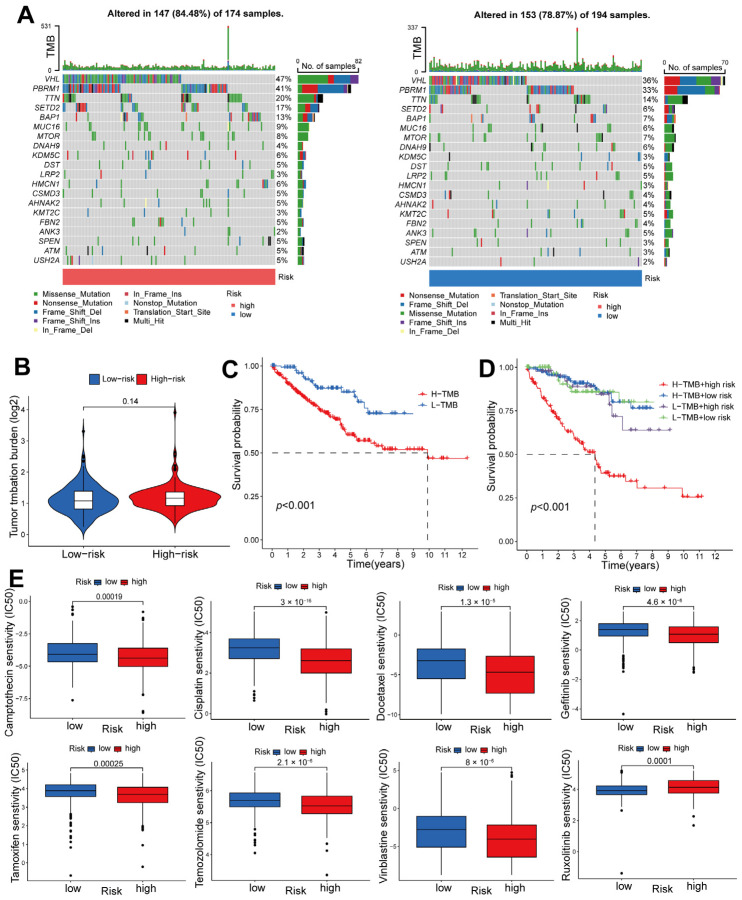
Risk-score-based characteristics of ccRCC tumor mutations and response to chemotherapy. (**A**) Waterfall diagrams displaying somatic genetic alterations in ccRCC individuals categorized as having a high (**left**) or low (**right**) risk score. (**B**) TMB values of patients at high risk and those at low risk. (**C**) An examination of the survival rates between groups with elevated and reduced TMB. (**D**) An analysis of the survival rates of the indicated four groups. (**E**) Predicted IC50 values to the indicated chemotherapy drugs comparing patients at high risk and those at low risk.

Based on the findings depicted in the waterfall chart in Figure 7A, it can be observed that among the two risk groups, a total of 20 genes exhibited the highest frequency of mutations. The mutation frequencies of these genes were notably higher in the patients at high risk as compared to those at low risk. Additionally, an examination of Figure 7B reveals that no significant disparity in Tumor Mutational Burden (TMB) was discernible between patients at high risk and those at low risk. Furthermore, it is worth mentioning that patients characterized by a higher TMB experienced a comparatively shorter OS period than did those with a low TMB (Figure 7C,D). The prediction of chemotherapy treatment response was accomplished through the calculation of predicted IC50 values. Consequently, it was observed that high-risk groups exhibited a more favorable predicted response to camptothecin, cisplatin, docetaxel, gefitinib, tamoxifen, temozolomide, and vinblastine, whereas low-risk groups demonstrated a superior predicted response to ruxolitinib (Figure 7E).

### 2.7. Immune Infiltration Pattern Variation between Different Risk Groups, and the Potential for Utilizing a Signature Related to CD8^+^ T Cells in Anti-PD-1/L1 Immunotherapy

**Figure 8:**
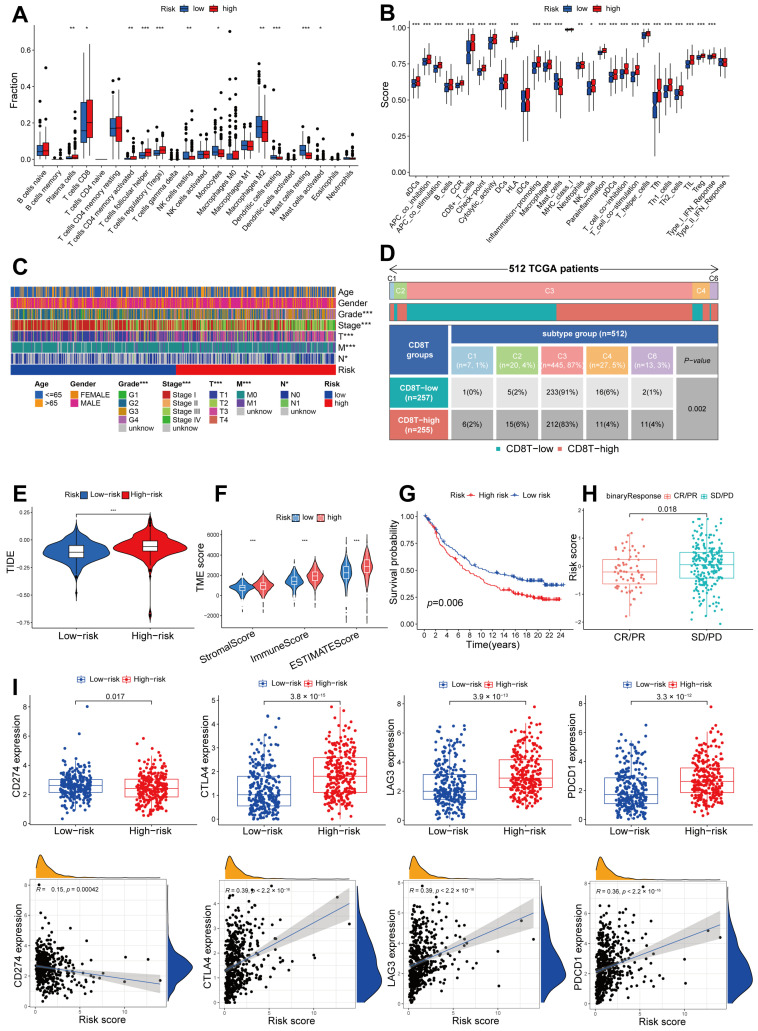
Immune infiltration landscape of different risk groups and potential of the CD8^+^ T-cell-related signature in anti-PD-1/L1 immunotherapy. (**A**) An algorithm based on CIBERSORT showing the fractions of 22 immune cells: * *p* < 0.05; ** *p* < 0.01; *** *p* < 0.001. (**B**) Scores for 29 immunotherapy-associated gene sets based on ssGSEA: * *p* < 0.05; ** *p* < 0.01; *** *p* < 0.001. (**C**) A comparison of the two risk subgroups based on clinical features: * *p* < 0.05; *** *p* < 0.001. (**D**) Correlation analysis between the risk model and immunophenotyping: *p* = 0.002 according to chi-square test. (**E**) An analysis of TIDE values in the patients at high risk or low risk: *** *p* < 0.001. (**F**) TME scores for the patients at high risk or low risk: *** *p* < 0.001. (**G**) The overall survival (OS) for IMvigor210 patients at high risk or low risk, treated with anti-PD-L1. (**H**) The risk scores of patients who achieved CR/PR or SD/PD (CR, Complete Response; PR, Partial Response; SD, Stable Disease; PD, Progressive Disease). (**I**) The expression of immune checkpoints and the Spearman correlation between their expression and risk score.

The immune infiltration patterns of ccRCC samples were characterized using the ‘CIBERSORT’ algorithm to determine the fractions of immune cells. Notably, the high-risk group exhibited a marked elevation in the presence of plasma cells, CD8^+^ T cells, memory activated CD4^+^ T cells, follicular helper T cells, and regulatory T cells. Conversely, the group with low risk displayed an abundance of inactive natural killer cells, monocytes, M2 macrophages, inactive dendritic cells, and inactive mast cells (Figure 8A). Afterwards, the ssGSEA technique was utilized to estimate scores for particular immune cells and functions in a single-sample gene set enrichment analysis. The analysis revealed significant disparities between the patients at high risk and those at low risk in relation to the majority of immune cells and functions (Figure 8B). To gain further insights, heatmap visualizations were utilized to investigate potential distinctions in clinical variables among the risk groups. Notably, the patients at high risk or low risk exhibited significant dissimilarities in clinical variables, with the exception of age and gender (Figure 8C). A recent study identified six immune subtypes across cancer tissue types and molecular subtypes. These subtypes are C1 (associated with wound healing), C2 (dominated by IFN-γ), C3 (characterized by inflammation), C4 (depleted of lymphocytes), C5 (immunologically silent), and C6 (dominated by TGF-β) [20]. In the subgroup characterized by high levels of CD8^+^ T cells, the prevalence of C1, C2, and C6 was higher, whereas C3 and C4 were more commonly found in the subgroup with low levels of CD8^+^ T cells (Figure 8D). An online tool was utilized to calculate the TIDE score, which forecasts the reaction to immunotherapy in patients at high risk or low risk. Our findings indicate that patients categorized as high risk exhibited notably elevated TIDE scores in comparison to those categorized as low risk (Figure 8E). Furthermore, the high-risk cohort demonstrated superior stromal scores, immune scores, and estimated scores compared to the low-risk cohort (Figure 8F). The two risk groups were analyzed for their response to anti-PD-L1 using the IMvigor 210 database. According to the KM curve analysis, the low-risk category in Imvigor 210 demonstrated a greater overall survival rate (Figure 8G). Additionally, individuals who achieved complete response (CR) or partial response (PR) exhibited a reduced risk score (Figure 8H), suggesting that the patients at low risk had a more favorable reaction to anti-PD-L1 treatment. Moreover, we assessed the prognostic capacity of the risk model for immune checkpoint inhibitor (ICI) response. The patients at high risk demonstrated increased expression of immune checkpoint molecules CTLA4, LAG3, and PDCD1, indicating that ICI treatment may be more effective for this group (Figure 8I).

### 2.8. Validation of the Expression of the Seven CD8^+^ T-Cell-Related Genes

**Figure 9:**
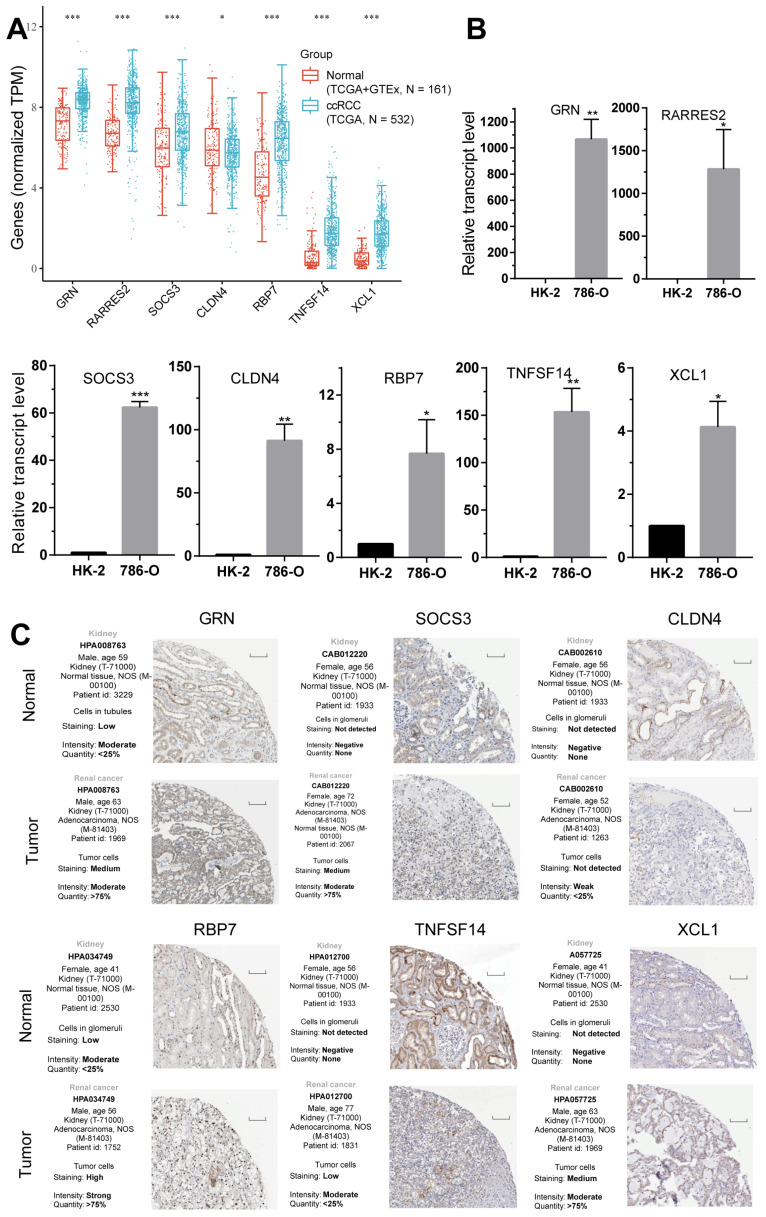
Evaluating the expression of 7 CD8^+^ T-cell-associated genes. (**A**) The expression distribution of the 7 CD8^+^ T-cell-associated genes in tumor tissues of ccRCC and normal tissues: * *p* < 0.05; *** *p* < 0.001. (**B**) qRT-PCR analysis for the expression of 7 CD8^+^ T-cell-associated genes in the 786-O ccRCC cell line and the HK-2 normal renal cell line: * *p* < 0.05; ** *p* < 0.01; *** *p* < 0.001. (**C**) Immunohistochemistry (IHC) was utilized to compare the expression of six proteins between normal and tumor tissues from the HPA. Scale bar = 100 µm.

To examine the expression of the seven CD8^+^ T-cell-related genes in ccRCC tumors, we utilized the TCGA and GTEx databases to conduct gene expression analysis. The findings revealed that, with the exception of *CLDN4*, the remaining six CD8^+^ T-cell-related genes displayed elevated expression levels in ccRCC tumors (Figure 9A). The mRNA expression of the seven signature genes in tumor and normal tissues was further validated using qRT-PCR. The results demonstrated that the mRNA expression of all seven genes was significantly elevated in ccRCC tumor tissues (Figure 9B). Additionally, the expression of these proteins was verified using the Human Protein Atlas (HPA) database. Figure 9C illustrates that the protein expression was notably higher in renal tumor tissues compared to normal tissue. These findings provide substantial evidence supporting the crucial involvement of these genes in ccRCC.

## 3. Discussion

A nephrectomy remains the most efficacious option for primary clear cell renal cell carcinoma (ccRCC), despite the fact that 20–40% of patients experience relapses and metastases following the surgery [21]. Given the growing availability of novel treatment modalities for ccRCC, there is an urgent requirement for biomarkers to monitor prognosis [22]. The tumor microenvironment of ccRCC exhibits infiltration by various immune cells, each exerting distinct effects on prognosis [23]. Notably, the presence of CD8^+^ T cells has been associated with unfavorable prognosis in ccRCC tumors, posing significant challenges for immunotherapy [24]. The enhancement of tumor immunotherapy could potentially be achieved through the recognition of key genes related to the infiltration of CD8^+^ T cells. Our research commenced by acquiring and processing scRNA-seq data in the GEO database. Through gene differential analysis, we obtained DEGs specific to CD8^+^ T cells. Subsequently, utilizing TCGA-KIRC cohort data, we conducted univariate Cox regression analysis on the DEGs to recognize prognostic genes. A random forest model was then constructed based on seven CD8^+^ T-cell-related genes (*RARRES2*, *SOCS3*, *TNFSF14*, *XCL1*, *GRN*, *CLDN4*, and *RBP7*), which were subsequently validated to exhibit favorable predictive accuracy. Consequently, a notable association was observed between the risk score and the immune profile, thereby serving as a prognostic indicator for the reaction to immunotherapy. Furthermore, the risk score exhibited correlations with multiple immune checkpoints. Finally, the confirmation of CD8^+^ T-cell-associated genes was conducted using qRT-PCR.

Chemerin (RARRES2), an adipokine and chemoattractant factor, has been implicated in obesity, inflammatory disorders, and cancer [25]. Enhanced chemerin expression within the TME of breast carcinoma impedes tumor growth by attracting NK and T cells [26]. In comparison to normal tissues, the expression of chemerin/RARRES2 is commonly down-regulated in various tumors [26]. However, contrary to previous studies, our research demonstrates that the expression of RARRES2 is elevated in ccRCC tumors in comparison to normal tissue. Therefore, further investigation is warranted to explore the potential role of RARRES2 in ccRCC. Additionally, SOCS3 acts as an inhibitor of cytokine signaling, governing the differentiation of CD4^+^ T cells and the development of CD8^+^ T cells, alongside its role in modulating innate immune cells and influencing tumorigenesis [27]. Aberrant methylation of *SOCS3*, a tumor suppressor gene, has been observed in several types of human cancer [28]. This contradicts our result that higher expression of SOCS3 was observed in ccRCC tumor. Previous studies have reported that tumor necrosis factor superfamily 14 (TNFSF14/LIGHT) has a role in the activation of T lymphocytes [29]. Additionally, it has been shown to have a significant impact on regulating antitumor immunity by stimulating T cell proliferation and inducing apoptosis in various tumor cells. [30]. Furthermore, NK cells release XCL1, which has been found to promote DC aggregation and CD8^+^ T cell proliferation in solid tumors [31]. However, the association of *GRN*, *CLDN4*, and *RBP7* with CD8^+^ T cell infiltration has not been reported. Therefore, further investigation is warranted.

The analysis of immune cell infiltration is of paramount importance in the assessment and management of diseases. This study involved a thorough examination of the immune landscape in patients in varying ccRCC risk groups. To explore the abundance and functions of immune cells, we utilized the CIBERSORT, ESTIMATE, and ssGSEA methodologies. Notably, the levels of immune cell infiltration, particularly CD8^+^ T cells, allowed for the identification of two distinct tumor phenotypes: “hot” and “cold”. These phenotypes correspond to favorable and unfavorable responses to T cell checkpoint inhibition, respectively [32]. Surprisingly, the high-risk group demonstrated a greater presence of plasma cells, CD8^+^ T cells, memory-activated CD4^+^ T cells, follicular helper T cells, and regulatory T cells, accompanied by a higher immune score, thus warranting classification as a “hot” tumor phenotype. Conversely, the low-risk group exhibited a higher abundance of resting NK cells, monocytes, M2 macrophages, resting dendritic cells, and resting mast cells, along with a lower immune score, indicating a “cold” tumor phenotype. Moreover, the high-risk patients exhibited elevated expression of the immune checkpoint molecules CTLA4, LAG3, and PDCD1. On the contrary, the patients at low risk demonstrated higher expression of the CD274 (PD-L1) immune checkpoint, suggesting a more favorable reaction to anti-PD-L1 treatment. Existing evidence suggests that higher PD-L1 expression is generally associated with enhanced effectiveness of immunotherapy [33]. This conclusion was further validated through analysis of the IMvigor 210 database. Additionally, the two groups demonstrated divergent responses to eight chemotherapy drugs, namely, camptothecin, cisplatin, docetaxel, gefitinib, tamoxifen, temozolomide, vinblastine, and ruxolitinib. Among them, camptothecin, a TOP1 inhibitor, and cisplatin have demonstrated sensitivity to ccRCC cell lines [34,35]. Considering that RCC is chemoresistant, we think that these different responses to chemotherapy drugs may have no clinical significance. The level of TMB has been recognized as a marker for assessing the effectiveness of immunotherapy [36]. Nevertheless, our research suggests that there is no substantial disparity in TMB among patients categorized as low or high risk. Conversely, the alteration frequency of *VHL*, *PBRM1*, *TTN*, and *SETD2* was relatively high in the patients at high risk compared to those at low risk. Mutations in *VHL* are among the most frequently observed in ccRCC, and it has been established that the suppression, removal, or methylation of *VHL* promotes tumorigenesis and cancer progression [37]. Mutations in the *PBRM1* gene have been shown to contribute to the development of tumors by inhibiting the ability of natural killer cells to clear tumor cells [38]. A deficit of *SETD2* promotes tumorigenesis through the activation of oncogenic transcription [39]. The examination of mutational signatures can aid in the more precise selection of immunotherapies for individual patients. Additionally, our study revealed that the expression levels of these seven signature genes were significantly elevated in ccRCC tumor tissues, as confirmed by qRT-PCR and the HPA database.

In this study, however, experimental studies were not conducted to validate the functions of the seven genes. Consequently, additional clinical trials will be required to substantiate the predictive capability of the risk model.

## 4. Materials and Methods

### 4.1. Data Acquisition

A total of 826 CD8^+^ T-cell-related DEGs were obtained from the Tumor Immune Single Cell Hub (TISCH) database using the KIRC single-cell dataset GSE159115 (http://tisch.comp-genomics.org (accessed on 21 April 2023)). Another 2533 immune-related genes were acquired from the InnateDB (https://www.innatedb.com/ (accessed on 13 February 2023)) and ImmPort (https://www.immport.org/home (accessed on 13 February 2023)) datasets. In order to obtain data for the ccRCC RNA-seq, including clinical and mutational data, the GDC Data Portal was used from the TCGA database (https://portal.gdc.cancer.gov/ (accessed on 21 April 2023)).

### 4.2. Construction of a Predictive Signature in TCGA

The ccRCC patients were randomly assigned to training and testing subgroups in a 1:1 ratio using the package “caret” (version 6.0-94) [40,41]. For the purpose of determining optimal prognostic genes, we used the “glmnet” (version 4.1-7) package to perform LASSO-Cox analysis, which is used to identify important predictors of survival outcomes and to reduce the number of predictors in the model [42]. CcRCC risk scores were calculated by combining normalized gene expression values with LASSO-Cox coefficients, following the formula below:

$$\mathrm{Risk}\;\mathrm{score}=\sum_{i=1}^{n}Coefi\times Expi$$

*Coefi* indicates the *i*th gene’s regression coefficient in LASSO-Cox, and *Expi* represents its mRNA expression value. The independent prognostic factors were validated using Kaplan–Meier (KM) graphs, receiver operating characteristic (ROC) curves, and univariate/multivariate Cox regression analyses. To assess the accuracy of our model, we compared our model with other signatures by the concordance index (C-index) and ROC analysis.

### 4.3. Analysis of Functional Enrichment

To comprehend the pathways and biological roles linked to the DEGs associated with CD8^+^ T cells, we conducted Gene Ontology (GO) and Kyoto Encyclopedia of Genes and Genomes (KEGG) analysis using the “clusterprofiler” (version 4.8.1) package [43,44].

### 4.4. Immunotherapeutic Sensitivity Analysis of the CD8^+^ T-Cell-Related Signature

ESTIMATE (version 1.0.13) and “CIBERSORT” (version 1.03, http://cibersort.stanford.edu/ (accessed on 22 April 2023)) analyses were used to estimate immune infiltration scores and relative proportions for 22 immune cells in the TCGA-KIRC database. “CIBERSORT” uses a support vector machine algorithm to assign each gene expression profile to a reference set of cell type profiles. Using the “GSVA” (version 1.48.2) package, 29 immune features were enriched using the ssGSEA approach [45]. An evaluation of half-maximal inhibitory concentrations (IC50s) for eight common chemotherapeutic drugs (camptothecin, cisplatin, docetaxel, gefitinib, tamoxifen, temozolomide, vinblastine, and ruxolitinib) was performed using the “pRRophetic” (version 0.5) package [46]. Briefly, the chemotherapeutic response for each sample was forecasted using the Genomics of Drug Sensitivity in Cancer (GDSC) database (https://www.cancerrxgene.org/ (accessed on 19 June 2023)), which is the largest publicly accessible pharmacogenomics database. The prediction procedure was executed through the utilization of the R package “pRRophetic”. The estimation of the IC50 for the samples was accomplished through ridge regression. All parameters were configured to their default values. In order to evaluate the tumor mutational burden (TMB), the “maftools” (version 2.16.0) package was used [47]. Patients with high and low risk were compared for the expression of CD274 (PD-L1), CTLA4, LAG3, and PDCD1 (PD1), which are commonly used as targets for immune checkpoint inhibitor (ICI) therapies. Tumor immune dysfunction and exclusion (TIDE) scoring was assessed in each ccRCC patient according to the instructions on the TIDE website (http://tide.dfci.harvard.edu/ (accessed on 24 April 2023)) [48]. The effectiveness of anti-PD-L1 immunotherapy was also evaluated by analyzing the IMvigor210 group [49].

### 4.5. qRT-PCR

Cell lines 786-O for ccRCC and HK-2 for normal cells were extracted using the TRIzol method (Invitrogen Co., Carlsbad, CA, USA). The above two cell lines were purchased from a commercial company (Zhong Qiao Xin Zhou Biotechnology Co., Ltd., Beijing, China). For cDNA synthesis, a High Capacity RNA-to-cDNA kit (Toyobo Co., Ltd., Osaka, Japan) was used. Supplementary Table S4 details the primers we used for qRT-PCR assays. *GAPDH* was used as the internal control. The results were analyzed according to the method detailed in a previous study [50].

### 4.6. Human Protein Atlas (HPA)

The HPA dataset (https://www.proteinatlas.org/ (accessed on 29 April 2023)) was used to examine gene expression in tumor and normal tissues by immunohistochemistry (IHC).

## 5. Conclusions

By conducting a thorough examination of both single-cell and bulk RNA sequencing data pertaining to ccRCC, a novel and resilient model was formulated and subsequently verified. This model is predicated on seven genes associated with CD8^+^ T cells and is poised to enhance comprehension of the immune characteristics inherent to ccRCC, facilitate prognostic predictions for patients afflicted with ccRCC, and offer valuable recommendations for the use of immunotherapy approaches.

## Figures and Tables

**Table 1:** Clinical characteristics of patients with ccRCC.

Covariates	Total (*n* = 533)	Test (*n* = 266)	Train (*n* = 267)	*p* Value
Age				0.5443
≤65	349 (65.48%)	178 (66.92%)	171 (64.04%)	
>65	184 (34.52%)	88 (33.08%)	96 (35.96%)	
Gender				0.6735
Female	188 (35.27%)	91 (34.21%)	97 (36.33%)	
Male	345 (64.73%)	175 (65.79%)	170 (63.67%)	
Grade				0.4805
G1	14 (2.63%)	7 (2.63%)	7 (2.62%)	
G2	229 (42.96%)	107 (40.23%)	122 (45.69%)	
G3	206 (38.65%)	106 (39.85%)	100 (37.45%)	
G4	76 (14.26%)	43 (16.17%)	33 (12.36%)	
Unknown	8 (1.5%)	3 (1.13%)	5 (1.87%)	
Stage				0.1334
Stage I	267 (50.09%)	128 (48.12%)	139 (52.06%)	
Stage II	57 (10.69%)	34 (12.78%)	23 (8.61%)	
Stage III	123 (23.08%)	56 (21.05%)	67 (25.09%)	
Stage IV	83 (15.57%)	48 (18.05%)	35 (13.11%)	
Unknown	3 (0.56%)	0 (0%)	3 (1.12%)	
T				0.2643
T1	273 (51.22%)	130 (48.87%)	143 (53.56%)	
T2	69 (12.95%)	41 (15.41%)	28 (10.49%)	
T3	180 (33.77%)	88 (33.08%)	92 (34.46%)	
T4	11 (2.06%)	7 (2.63%)	4 (1.5%)	
M				0.4739
M0	422 (79.17%)	208 (78.2%)	214 (80.15%)	
M1	79 (14.82%)	43 (16.17%)	36 (13.48%)	
Unknown	32 (6%)	15 (5.64%)	17 (6.37%)	

## Data Availability

The datasets used and/or analyzed during the current study are available from the TCGA, GEO, and TISCH databases.

## Supplementary Materials

The following supporting information can be downloaded at: https://www.mdpi.com/article/10.3390/ijms241813729/s1.
